# The Influence of Expanded Glass and Expanded Clay on Lightweight Aggregate Shotcrete Properties

**DOI:** 10.3390/ma15051674

**Published:** 2022-02-23

**Authors:** Algirdas Augonis, Ernestas Ivanauskas, Vytautas Bocullo, Aras Kantautas, Danutė Vaičiukynienė

**Affiliations:** Faculty of Civil Engineering and Architecture, Kaunas University of Technology, Studentu St. 48, LT-51367 Kaunas, Lithuania; algirdas.augonis@ktu.lt (A.A.); ernestas.ivanauskas@ktu.lt (E.I.); vytautas.bocullo@ktu.lt (V.B.); aras.kantautas@ktu.lt (A.K.)

**Keywords:** lightweight aggregate shotcrete, expanded glass, expanded clay, cement supplementary materials, thermal conductivity coefficient

## Abstract

In the construction industry, the selection of sustainable materials leads to a movement towards more sustainable construction. In this study, lightweight aggregate shotcrete based on expanded glass (EG) and expanded clay (EC) is investigated. The goal of the study is to determine the influence of EG and EC inclusion on the properties of shotcrete. Ordinary Portland cement (OPC) powder with supplementary cementitious materials, such as silica fume and ground glass waste, are used as binders. The mechanical, physical and morphological properties, as well as the mineral and oxygen compositions, are obtained through compressive and flexural strength tests, thermal conductivity measurements, scanning electron microscopy with energy dispersive X-ray spectrometry (SEM–EDX), X-ray diffraction (XRD) and X-ray fluorescence (XRF) analysis. In this study, the mechanical, physical and thermal properties and waste utilization as cement supplementary materials are balanced. The shotcrete samples show that a density of 790 kg/m^3^ had a good thermal performance (thermal conductivity coefficient of 0.174 W/(m·K)) with the sufficient compressive strength of 6.26 MPa.

## 1. Introduction

To date, concrete is the most popular construction material with an annual production of approximately 10 billion m^3^ [1]. Due to the popularity of concrete, there are many technologies for building concrete structures. One of these rapidly emerging technologies is shotcrete (sometimes referred to as gunite, spraycrete or sprayed concrete). Shotcrete is a cement-based mixture that is sprayed at high velocity towards the target surface [2]. Because the construction industry in recent years has been increasing its sustainability, focusing on environmentally friendly technology, recycling, eco-friendly materials, and more sustainable construction materials are needed. So, it is important to create a more sustainable shotcrete.

One of the ways to increase the sustainability of shotcrete and concrete in general is by replacing aggregates by recycled and lightweight materials.

Normally, natural aggregates compose around 70–80% of concrete volume. The excavation of natural aggregates for concrete production harms the environment [2]. Concrete with lightweight aggregates can also reduce the thermal conductivity of the concrete, allowing heat to be preserved. Usually, a wide range of materials can be applied in the production of lightweight concrete, such as expanded glass (EG) aggregate, expanded clay (EC), plastic bottles, and silica aerogel, but with these, the materials’ strength decreases rapidly, so the application of these materials has its limitations regarding mechanical properties [3].

EC can be used in lightweight aggregate concrete production, due to the high technical properties and numerous advantages when compared with other industrial raw materials. The EC aggregate is a type of artificially produced lightweight aggregate that is manufactured by expanding natural clay at approximately 1200 °C in a rotary kiln [4]. The EC aggregate has great compressive strength compared to other lightweight aggregates [5]. Furthermore, EC aggregates provide better conditions for cement hydration, which reduces water absorption [6]. The authors of this study stated that the pre-wetting of EC aggregates prior to mixing had a positive effect on the additional water in the system. Due to this, internal curing mechanical properties were improved, and the pore structure of the material was densified.

The expanded glass (EG) is a type of ultra-lightweight aggregate capable of enhancing various concrete characteristics. It can partially replace natural aggregates and can change the properties of the concrete [7]. EG has been the subject of research due to its characteristics, such as stiffness, lightness, and compressive strength and, furthermore, it normally requires less energy for its production compared to conventional materials [2]. Currently, numerous amounts of waste glass are collected from various industrial plants and then dumped. The EG aggregate is prepared from waste glass from various industries [7]. By incorporating EG into concrete, the amount of glass waste and natural aggregate excavation are reduced and concrete properties can be changed, making it more sustainable.

Various investigations have been carried out on the effect of EG on the properties of concrete. EG has gained attention as a promising material for sustainable lightweight concrete. A drastic decrease in compressive strength may not be the case when EG is used. Sharma et al. [7] investigated the influence of the variation in particle size distribution on the properties of concrete. It was concluded that the smaller the size of the aggregate particle, the more the compressive strength will develop and, on the contrary, the bigger the size of the aggregate particles, the less the compressive strength will develop. This is due to the smaller size and smooth surface of the EG, which improves the bonds between the cement matrix and the aggregate particles. Additionally, it densifies the microstructure of the concrete by acting as a filler for the pores inside the concrete [7]. Chung et al. [8] concluded that lightweight aggregate concrete could be more useful to mechanical operations by reducing insulation loss. The compressive strength of samples with crushed waste glass increased by about 20% compared with the samples in which natural sand was used. The highest compressive strength of samples with alternative lightweight aggregates was over 36 MPa with a thermal conductivity coefficient 0.6 W/(m·K).

EG aggregates, because of their cellular and porous structure, significantly reduce the density and thermal conductivity of the lightweight concrete: the air thermal conductivity within the pores is approximately 0.024 W/(m·K), so it has a significant effect on thermal insulation [9]. Real et al.’s [10] research has shown an average reduction of 0.6% in thermal conductivity when porosity is increased by 1%. Uysal et al. [11] concluded that, by replacing 25–100% of the natural aggregate by pumice lightweight aggregate, the thermal conductivity and density of the concrete decreased by 46% and 40%, respectively. Additionally, due to its lower density, EG could improve acoustic comfort: the lightweight porous EG concrete has sufficient noise absorption [12].

On the other hand, there are some issues. Bumanis et al. [13] noted that lightweight concrete with EG granules are threatened by alkali silica reaction (ASR) between the EG and alkalis in Portland cement. The research has shown that even concrete produced with low alkali Portland cement (e.g., Aalborg CEM I 52.5R) underwent a high relative expansion (0.16%), which does not satisfy RILEM AAR-2 recommendations. Additionally, ASR significantly affected concrete strength: flexural strength was reduced from 62% to 73%, meanwhile compressive strength was reduced from 23% to 43%, compared to water-cured specimens. However, choosing the right Portland cement with very low Na_2_O_eq_ could reduce ASR risks.

Shotcrete, sometimes referred as sprayed concrete in the literature, is a cement-based mixture that is sprayed at a high velocity towards the target surface. Currently, very few investigations have been conducted to determine the thermal properties of shotcrete, especially when a lightweight aggregate is included [14].

It can be concluded that EG and EC improves the properties of concrete—when natural aggregates were partially or fully substituted with lightweight aggregates, such as EG or EC, a lighter concrete with excellent mechanical and slump properties was obtained [2]. The aim of this study is to investigate the application of EG and EC as a replacement of natural aggregates in the production of a mixture for shotcrete. The powder of silica fume and ground glass waste was incorporated in the lightweight aggregate shotcrete as cement supplementary materials.

Currently, it is important to use cementitious materials in concrete systems. These materials lead to a significant reduction in CO_2_ emissions during the production of Portland cement. A large number of studies have been conducted on ground glass and silica fume and/or mixtures as cementitious materials (Table 1).

A traditional type of cementitious material is silica fume, which could improve the strength and durability properties of cement systems. Due to the fine particle size of silica fumes, the reactivity is generally larger compared with ground glass waste. This reactivity is closely related to the increase in the compressive strength for mortar or concrete [15,16,17,18].

In cement systems, glass waste ground very finely acted as a pozzolanic material and for that reason it is possible to substitute some amount of Portland cement with ground glass waste. By comparing two types of cementitious materials, such as ground glass waste and silica fume, mortar or concrete containing ground glass waste exhibited a similar or slightly lower compressive strength [15,16,17,18,19].

The highest compressive strength was detected in mixtures with a combination of silica fumes and ground glass powder or expanded glass [18,19]. In some cases, the values of compressive strength reached the values of high-strength concrete [19]. Vaitkevičius et al. [20] determined that ground glass waste is similar to a contender for silica fumes, which had the positive development of compressive strength.

Currently, very few investigations have been performed to determine the thermal properties of shotcrete, especially when lightweight aggregate is included [14]. So, the aim of this research is to determine the influence of the lightweight aggregates, EG and EC, on thermal conductivity, compressive strength, water absorption, porosity, macrostructure and mineral composition, and to analyze the correlation between these properties.

## 2. Materials and Methods

For the production of the lightweight aggregate shotcrete, the commercial ordinary Portland cement (OPC) Rocket, Swedish, CEM I 42.5R type, was used as the main binder. The oxygen composition of OPC is shown in Table 2. The superplasticizer of Sika^®^ ViscoCrete^®^ D-187 (Sika Services AG, Zürich, Switzerland), was incorporated for the reduction in the water amount in the mixtures.

The powder of silica fume and ground glass waste was incorporated in the lightweight aggregate shotcrete as cement supplementary materials. These two cement supplementary materials were chosen because they can improve the strength properties of shotcrete and substitute a portion of the expensive Portland cement, as stated by Mehta et al. [21].

The oxide composition of these materials is presented in Table 2. In silica fumes, SiO_2_ dominated and it consisted of more than 97%. Other oxides did not exceed 1%. Three types of oxides, SiO_2_, Na_2_O and CaO, consisted of the largest part in the ground glass waste.

**Figure 1:**
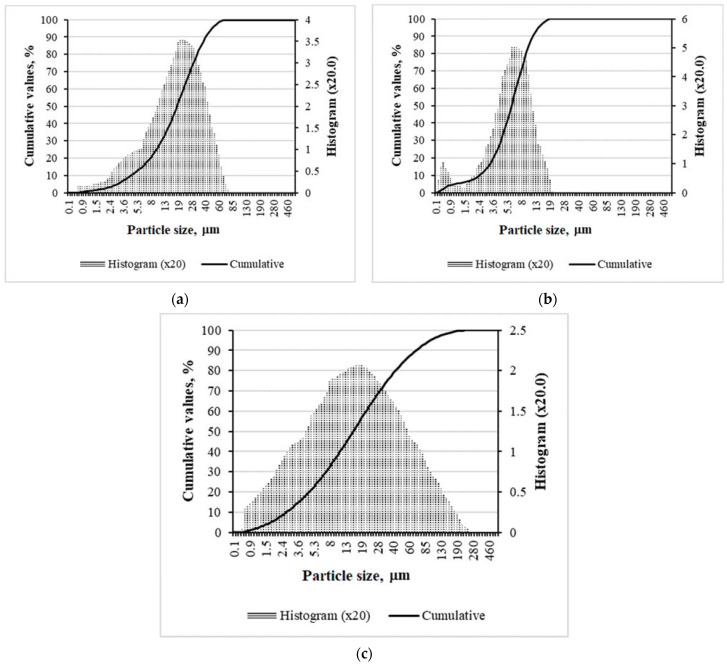
The particle size distributions of: (**a**) OPC; (**b**) silica fume; and (**c**) ground glass.

The particle size distributions of OPC are shown in Figure 1a. The particles of OPC varied in the range between 1.5 µm and 60 µm and average particles had the size of d = 21.66 µm. The particles of silica fume had a more narrow interval (from 1.5 µm to 19 µm) than particles of OPC and the size of average particles was 6.81 µm (Figure 1b). The particles of ground glass waste belong to a wide range of distribution from 0.9 µm to 190 µm (Figure 1c), and the size of the average particles consisted of d = 28.45 µm.

In order to produce lightweight aggregate shotcrete, it is necessary to include lightweight aggregates. In the mixtures of this shotcrete, two types of artificial lightweight aggregate were used for the production of the shotcrete mixtures: the expanded glass from JSC Stikloporas (Lithuania) of 0.125/0.25, 0.25/0.5, 0.5/1, 1/2, 2/4, 4/8 fractions and SC Palemono keramika (Lithuania) expanded clay of 4/8 fraction. The values of the loose bulk density were evaluated according to EN 1097-3, the coefficient of thermal conductivity λ according to EN 12939, bulk crushing resistance according to EN 13055, and water absorption according to EN 1097-6.

The high-quality shotcrete was closely related to the main properties of lightweight aggregates: loose bulk density, bulk crushing resistance and water absorption. According to Table 3, the highest water absorption (19.3%) had Mix 3 (expanded clay) and the lowest water absorption (9.2%) had Mix 2 (expanded glass) with the same fraction of 4/8 mm. Similar values of water absorption for expanded clay and expanded glass were determined by Vijayalakshmi et al. [22] and Yu et al. [23], respectively. The best mechanical properties, such as bulk crushing resistance, had Mix 3 with expanded clay (4/8) and it was 2.5 times higher than the particles of expanded glass (4/8). The higher particles of lightweight aggregates led to a lower bulk density, bulk crushing resistance and thermal conductivity [24].

**Figure 2:**
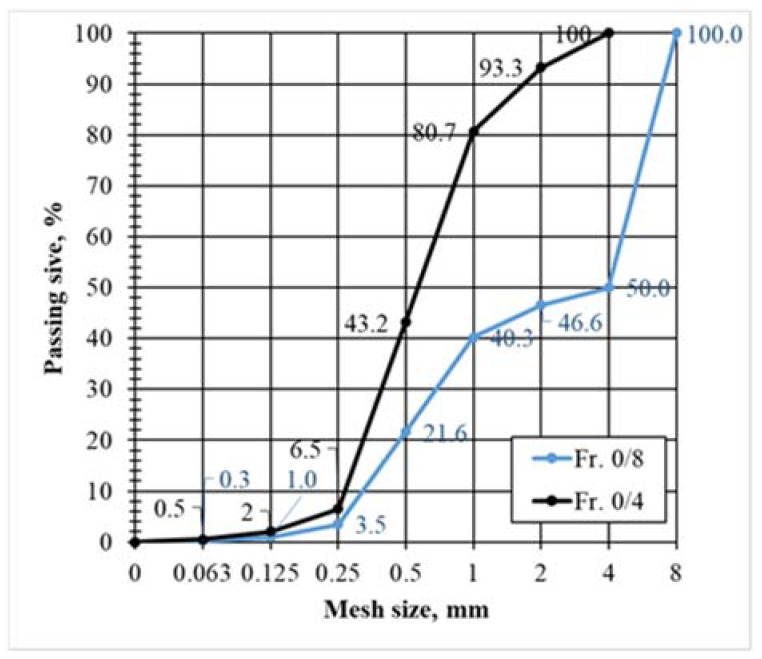
The grain size distribution of lightweight aggregate blends for lightweight aggregate shotcretes. The black curve is Mix 1, and the blue curve is Mix 2 and Mix 3.

Two types of lightweight aggregate blends were prepared for the experimental studies of lightweight aggregate shotcretes (Figure 2).

In Mix 1 and Mix 2, only expanded glass aggregates of different fractions were incorporated (Table 4). For mixture 3, expanded glass aggregates and expanded clay aggregates were used together. The right choice of granulometry for shotcretes affects the main properties, such as the strength, density, and thermal conductivity of the materials. In the preparation of Mix 1–Mix 3, the spaces between aggregate particles were considered. The lower number of spaces led to lower fine fractions and Portland cement. In order to fill spaces maximally, the required number of aggregates was selected using the theory of dense packing [25].

The samples of lightweight aggregate shotcrete were produced in accordance with the standard EN 196-1. The samples were formed in 40 × 40 × 160 mm molds and after 24 h demolded and cured at 20 °C in water for a period of 28 days. After 28 days, the strength of samples was tested, and its micro-structure was analyzed. The density of the samples of lightweight aggregate shotcrete was determined according to EN 12390-7. The compressive and flexural strength was determined according to EN 12390-3 and EN 12390-5, respectively. The samples were tested for strength after 28 days using the hydraulic press of ToniTechnik 2020. The porosity (total, open and close) of samples was determined by the density of samples, according to the GoSt 12730.4-78 standard. Each of the values of strength and density were the average of three samples.

The mineral composition of hydrated samples was determined by using XRD analysis after 28 days. This analysis was performed on the D8 Advance diffractometer (Bruker AXS) operating at a tube voltage of 40 kV and tube current of 40 mA. The X-ray beam was filtered with a Ni 0.02 mm filter to select the CuK_α_ wavelength. Diffraction patterns were recorded in a Bragg–Brentano geometry using a fast-counting detector Bruker LynxEye based on silicon strip technology. The samples were scanned over the range 2θ = 3–60° at a scanning speed of 6 min^−1^ using a coupled two theta/theta scan type.

X-ray fluorescence (XRF) analysis was used for the evaluation of chemical oxide compositions. X-ray fluorescence spectroscopy (XRF) were conducted on a Bruker X-ray S8 Tiger WD spectrometer equipped with an Rh tube with energy of up to 60 keV.

The microstructure of hardened lightweight aggregate shotcrete was studied by scanning electron microscope. A high-resolution scanning electron microscope FEI Quanta 200 FEG with a Schottky field emission gun (FEG) was used for the research. For microscopy analysis, a “Ceti Stereo-Steddy” microscope was used. Pictures were taken with a 21 MP digital camera. The chemical compositions of contact zone for expanded glass and hardened cement paste were investigated by an energy-dispersive X-ray spectrometer (EDX) with a silicon type drift droplet detector.

The particle size distribution of the slag and phosphogypsum was determined using a laser particle size analyzer Cilas 1090.

For the determination of the thermal conductivity (coefficient λ), the samples were prepared according to the standard EN 12667. In this case, a heat flux meter with the steady-state hot box approach was used [26]. Three 300 × 300 × 50 mm size samples of each composition were prepared for the measurement. These samples were dried until constant weight.

## 3. Results

After the first 7 days, Mix 3, which contained EC coarse 8/16 aggregate, showed the largest compressive strength, reaching 12.99 MPa (Table 5). After 28 days of curing, the significant compressive strength gain was only observed for Mix 3, at 9.84 MPa, which is a 58.3% gain compared to the 7 day strength. Meanwhile, Mixes 1 and 2 reached lower compressive strength values after 28 days, 8.49 MPa and 6.26 MPa, respectively, and the majority of the compressive strength developed within the first 7 days. For the following 21 days, the gain was only 4.4% and 20.0%.

The thermal conductivity test showed that the best thermal insulator is Mix 2 (0.174 W/(m·K)); naturally, this mix possessed the lowest bulk density at 790 kg/m^3^. Mix 3 had the highest thermal conductivity coefficient, 0.322 W/(m·K) (Table 5). It is worth mentioning that Mix 3 had an EG 4/8 fraction replaced by EC of the same fraction, which resulted in higher density and compressive strength, but reduced the insulating properties.

**Figure 3:**
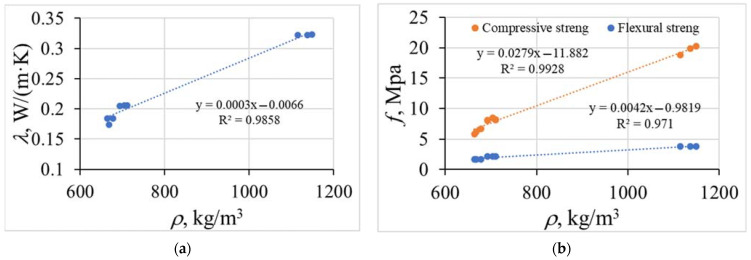
The relation between densities *ρ* of the dry samples with: (**a**) thermal conductivity coefficient *λ*; (**b**) mechanical strength *f*.

From the results of these tests, it is clear that the incorporation of lightweight aggregates into the concrete matrix can reduce thermal conductivity at the expense of compressive strength. All data were expressed as the average value of triplicate measurements. One sample was used for one measurement. Standard deviations did not exceed 5% for the values. As seen in Figure 3, there is a strong correlation between density and thermal conductivity and compressive strength. The relation between thermal conductivity can be described as:

*λ* = 0.0003*ρ* − 0.0066, (1)

where *ρ* represents the thermal conductivity coefficient W/(m·K) and *ρ* is the bulk density, kg/m^3^.

The relation between density and mechanical strength can be described as:

*f*_c_ = 0.0279*ρ* − 11.882, (2)

*f*_fl_ = 0.0042*ρ* − 9.819, (3)

where *f*_c_, *f*_fl_ represent the compressive and flexural strength (MPa), respectively, and *ρ* represents the bulk density, kg/m^3^.

The R^2^ of Equation (2) is 0.99, which means that 99% of the calculated compressive strength results match real values. The R^2^ of Equation (3) is 0.97, which means that 97% of the calculated flexural results match real values. Other factors accounted for 1% and 3%.

A similar relationship of density and compressive strength has been published by Adhikary et al. [24]. They stated that the lightweight concrete with EG aggregate reached up to 22 MPa of compressive strength and up to 3 MPa of flexural strength with a density of 1000 kg/m^3^. The compressive strength of concrete decreases with the incorporation of EG aggregates due to higher porosity and lower mechanical strength.

**Figure 4:**
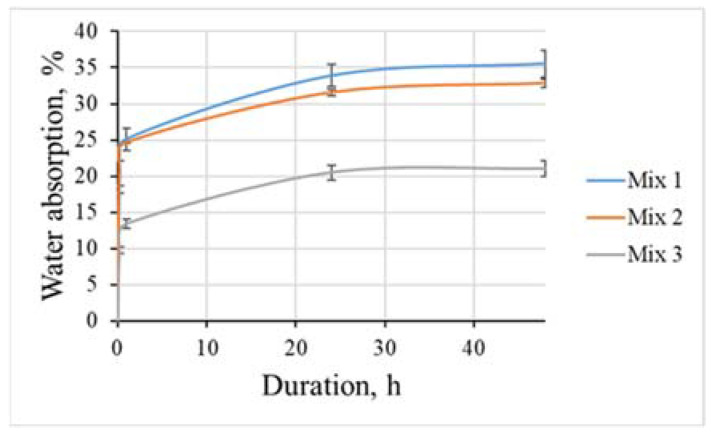
The kinetics of water absorption for lightweight aggregate shotcrete samples.

Water absorption is very important to thermal insulation materials because it changes the thermophysical properties. The lowest water absorption, 21.1%, was observed in the Mix 3 sample, which had the highest density and contained coarse EC aggregates (Figure 4). The Mixes 2 and 3 within the first 15 min showed very similar water absorption rates, 25.1% and 24.6%, respectively, but in the later stages, it was clear that the Mix 1 sample absorbed more water, as, after 48 h, it reached 35.5%, and the Mix 2 sample reached 33.6%. Even if Mix 1 absorbs more water than the Mix 2 sample, it is clear that there is a significant water absorption difference in the cases when the EG lightweight aggregate is used and when EC aggregate is used as a coarse lightweight aggregate.

**Figure 5:**
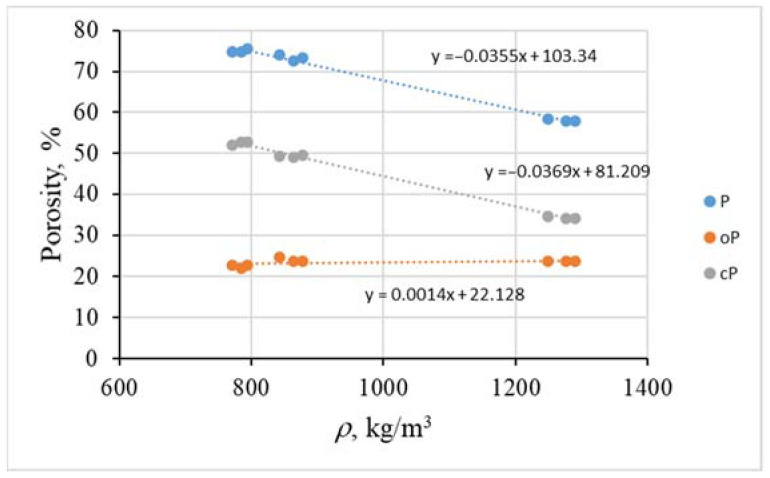
The porosity of lightweight aggregate shotcrete samples. P—total porosity; oP—open porosity; cP—closed porosity.

In the shotcrete samples, the amount of different types for lightweight aggregates influenced the porosity of the samples (Figure 5). The density of the lightweight aggregate shotcrete samples had a significant influence on the total and close porosity. By increasing the density of samples the close porosity of them decreased. A different situation can be observed in the case of the open porosity, which almost does not change with the increase in the density of the samples. It was in the range of 22.14% from 24.73%. Mix 2 had the highest total (74.9%) and close porosity (49.4%) with the lowest density. It was determined that the increase in total porosity is related to the increase in close porosity, while open porosity is almost stable.

**Figure 6:**
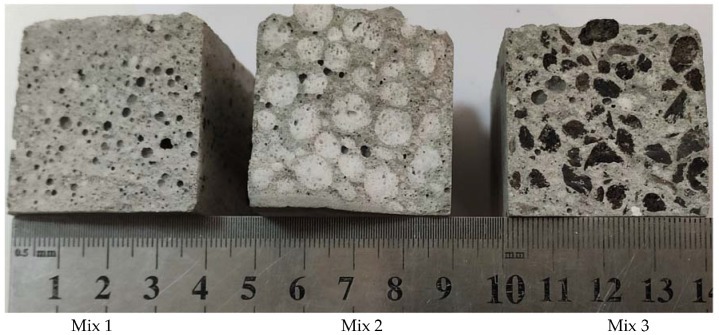
The macrostructure of the lightweight aggregate shotcrete samples.

According to the macrostructure analysis (Figure 6), the lightweight aggregates are evenly distributed, and this distribution is important to the thermal and mechanical characteristics. In addition, during the sample formation process, the closed pores (up to 1 mm in diameter) are formed, which improved the thermal insulation properties of the samples. This type of pore was detected in all samples, but the Mix 1 sample had the largest number.

**Figure 7:**
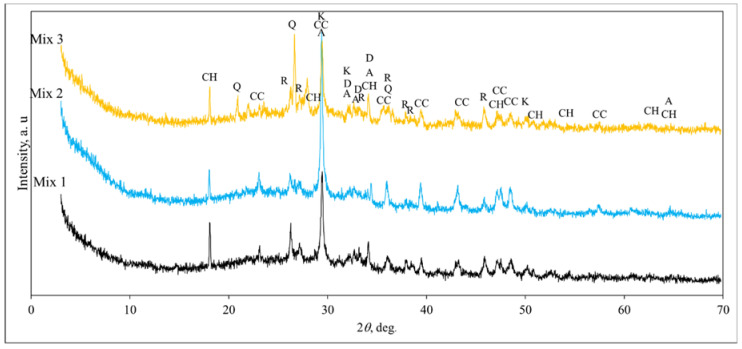
X-ray diffraction patterns of hardened shotcrete samples containing the lightweight aggregate after 28 days of hydration: CH—portlandite Ca(OH)_2_ (44–1481); A—alite Ca_54_MgAl_2_Si_16_O_90_ (13–272); D—larnite Ca_2_(SiO_4_) (83–461); K—calcium silicate hydrate Ca_1.5_Si O_3.5_∙x H_2_O (33–306); CC—calcite CaCO_3_ (72–1651); Q—quartz SiO_2_ (78–2315); and R—aragonite CaCO_3_ (71–2396).

The PDF-2 database was used for the identification of XRD diffraction peaks [27]. Figure 7 presents the XRD diffraction of the shotcrete samples. The mineral composition of all investigated samples is similar for all tree samples. In these samples, portlandite, alite, larnite, calcium silicate hydrate, aragonite and calcite were detected. These minerals were formed during the hydration of OPC with cement supplementary materials. In the Mix 3 sample, a quartz phase was detected as well. This mineral could be from the expanded clay. Abdelfattah et al. [28] examined the mineral phase of quartz in the lightweight expanded clay aggregates before and after heat-treatment.

**Figure 8:**
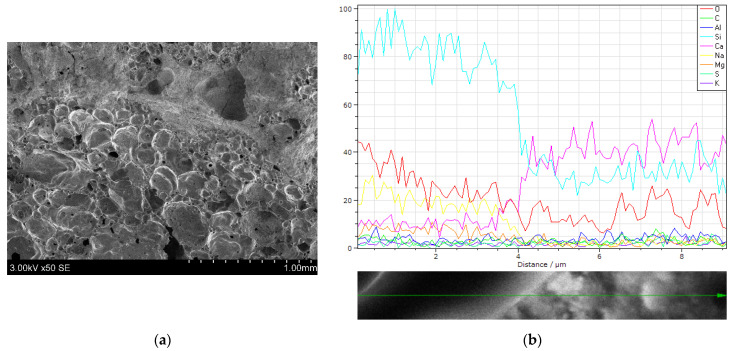
SEM images of the hardened cement paste samples containing expanded glass (**a**) and the elemental composition of transition zone for EG and hardened cement paste, according EDX analysis (**b**).

The transition zone between the expanded glass and hardened cement paste was investigated (Figure 8). This transition zone is difficult to detect because cement paste penetrates the aggregate due to its porous character (Figure 8a). From Figure 8b, it is revealed that the elemental composition of this composite material changes according to the analyzed place. The EDX analysis shows that silicon, oxygen and sodium dominated in the area of the expanded glass. A different chemical composition was detected in the matrix of the hardened cement paste. A lower amount of silicon, sodium and oxygen and a significantly higher amount of calcium were detected in the matrix. The other chemical elements did not change significantly. Similar EDX investigation results were also observed Bumanis et al. [13]. The minerals of the cement paste could be incorporated in the aggregates of the expanded glass.

As seen in Table 5, the density of hydrated samples is in the range of 790–1247 kg/m^3^ and the compressive strength is in the range of 6.26–19.84 MPa. The values of density are related to the values of compressive strength and both of them could be attributed to the porous microstructure of samples and transition zone for lightweight aggregates and hardened cement paste. The lightweight aggregates had an effect on the main properties of the shotcrete as well. The higher bulk crushing resistance of the expanded clay (2.8 MPa) led to the higher mechanical properties of shotcrete samples compared with the lower bulk crushing resistance of the expanded glass (1.1 MPa). A slightly higher density (800–2100 kg/m^3^) and significant lower values of compressive strength (1.16–4.26 MPa) were detected by Liu et al. [14].

In the shotcrete samples, the use of lightweight aggregate mixtures of different nature, EG and EC, successfully affected the main properties of the samples. According to the density (668–1137 kg/m^3^) after drying at 100 °C, these shotcretes could be assigned to the low density type, according to LST EN 206-1.

With the growing popularity of shotcrete, the knowledge of how lightweight aggregates influence the overall characteristics of concrete can show the way to more sustainable constructions. Knowing the advantages and disadvantages of the concrete of the materials, such as EG and EC, can clarify where this type of technology can be used. In our case, the relation between the aggregates used in the mixtures and their properties was determined, i.e., the relation between thermal conductivity and mechanical properties, such as compressive strength. The suggested sprayed concrete or shotcrete could be used as a coating material on different surfaces.

## 4. Conclusions

By using lightweight aggregates and by incorporating a water reducer with cement supplementary materials (ground glass waste and silica fume), it is possible to reach 19.8 MPa of compressive strength after 28 days of hydration. This strength was influenced by a compact transition zone between the expanded glass and the hardened cement paste, where the cement paste penetrates the aggregate of the expanded glass according to EDX analysis. The main properties of the lightweight aggregates were also influential. The relation between the compressive and flexural strength, density, and porosity with the thermal conductivity was examined. Finally, the combinations of lightweight aggregates and the composition of the binder produced a shotcrete with a relatively low density of 790 kg/m^3^, a thermal conductivity coefficient of 0.174 W/(m·K) and a compressive strength that reached 6.26 MPa. Therefore, this shotcrete has potential in construction materials’ industry.

## Figures and Tables

**Table 1:** The compressive strength of mortar with silica fumes and ground waste glass as cementitious materials.

Cementitious Materials	Compressive Strength, MPa (Mortar)	Specific Surface Area, m^2^/kg
Silica fume [15]	60	
Ground waste glass	53	400
Ground waste glass	57	600
Ground waste glass [16]	28–62	2.2–540
Silica fume [17]	52	
Ground waste glass	42–47	
Silica fume [18]	28–33 *	<75 μm
Glass powder	27–28 *	
Mixture of silica fume and ground waste glass	29–35 *	
Mixture of expanded glass and silica fume [19]	40–101	0.5–1 mm

*—concrete.

**Table 2:** Chemical oxide compositions and physical properties of ordinary Portland cement, silica fumes and ground glass waste, % (according to X-ray fluorescence (XRF) analysis).

Oxide	OPC	Silica Fume	Ground Glass Waste
SiO_2_	21	97.55	71.87
Al_2_O_3_	3.9	0.82	1.64
Fe_2_O_3_	2.9	0.03	0.75
La_2_O_3_	0.06	-	-
TiO_2_	0.03	-	0.04
MgO	2.3	0.14	2.11
CaO	66	0.38	9.76
Na_2_O	0.16	-	12.58
SO_3_	3.2	0.07	0.09
P_2_O_5_	0.15	-	0.02
K_2_O	0.24	0.11	0.79
Cl	0.06	-	-
Other	-	0.9	0.35
Bulk density, kg/m^3^	1236	421	1652
Specific density, kg/m^3^	3122	2538	2515
Surface area (by Blaine), m^2^/kg	350.0	-	334.0

**Table 3:** The physical and mechanical properties of lightweight aggregates.

Aggregate (Fraction, mm)	Loose Bulk Density, kg/m^3^	Thermal Conductivity Coefficient λ, W/(m·K),	Bulk Crushing Resistance, MPa	Water Absorption, %
Expanded glass (0.125/0.25)	375	-	-	10.2
Expanded glass (0.25/0.5)	346	0.0768	2.4	14.8
Expanded glass (0.5/1)	308	0.0716	2.1	17.9
Expanded glass (1/2)	234	0.0661	2.0	18.2
Expanded glass (2/4)	205	0.0636	1.3	15.1
Expanded glass (4/8)	158	0.0663	1.1	9.2
Expanded clay (4/8)	477	0.1023	2.8	19.3

**Table 4:** The composition lightweight aggregate shotcrete for a cubic meter (kg/m^3^).

Initial Materials	Mix 1	Mix 2	Mix 3
CEM I 42.5 R	408	360	408
Silica fume	45.3	40	45.3
Ground glass waste (0/0.125)	13.3	5.9	6.7
Expanded glass (0.125/0.25)	20.5	9.1	10.3
Expanded glass (0.25/0.5)	167.3	73.8	83.7
Expanded glass (0.5/1)	171.1	75.47	85.53
Expanded glass (1/2)	57.5	25.35	28.7
Expanded glass (2/4)	30.5	13.48	15.28
Expanded glass (4/8)	-	201.18	-
Expanded clay (4/8)	-	-	686.57
Superplasticizer	5.24	4.63	5.24
Water *	448	324	439

* The amount of water obtained for the F5 class consistency of pastes were determined experimentally.

**Table 5:** Samples’ physical and mechanical properties.

Mixes	Bulk Density, kg/m^3^		Thermal Conductivity Coefficient, W/(m·K)	Compressive Strength, MPa		Flexural Strength, MPaAfter 28 Days
	Fresh Mix	Hydrated		After 7 Days	After 28 Days	
1	909 ± 11	852 ± 11	0.2061192 ± 0.000393	8.24 ± 0.11	8.49 ± 0.09	2.20 ± 0.08
2	854 ± 9	790 ± 10	0.1737713 ± 0.000367	5.32 ± 0.10	6.26 ± 0.12	1.67 ± 0.10
3	1289 ± 13	1247 ± 15	0.3227247 ± 0.000461	12.99 ± 0.13	19.84 ± 0.11	3.81 ± 0.12
